# Exploring the use of research evidence in health-enhancing physical activity policies

**DOI:** 10.1186/s12961-015-0047-2

**Published:** 2015-10-13

**Authors:** Riitta-Maija Hämäläinen, Arja R. Aro, Ien van de Goor, Cathrine Juel Lau, Mette Winge Jakobsen, Razvan M. Chereches, Ahmed M Syed, On behalf of the REPOPA Consortium

**Affiliations:** National Institute for Health and Welfare, Helsinki, Finland; Unit for Health Promotion Research, Institute of Public Health, University of Southern Denmark, Niels Bohrs Vej 9, 6700 Esbjerg, Denmark; Tranzo, Scientific Center for Care and Welfare, Tilburg University, Postbus 90153, 5000 LE Tilburg, The Netherlands; Research Centre for Prevention and Health (RCPH), Capital Region of Denmark, Ndr. Ringvej 57, Afsnit 84/85, 2600 Glostrup, Denmark; Center for Health Policy and Public Health, Babes-Bolyai University, Pandurilor 7, Cluj-Napoca, Romania; NHS England, 80 London Road, London, SE1 6LH UK

**Keywords:** Evidence-informed policymaking, Health-enhancing physical activity, Policy, Research evidence

## Abstract

**Background:**

The gaps observed between the use of research evidence and policy have been reported to be based on the different methods of using research evidence in policymaking by researchers and actual policymakers. Some policies and policymaking processes may therefore be particularly well informed by research evidence compared to others. The aims of the present article are to explore the use of research evidence in health-enhancing physical activity (HEPA) policies, identify when research evidence was used, and find what other types of evidence were employed in HEPA policymaking.

**Methods:**

Multidisciplinary teams from six EU member states analysed the use of research evidence and other kinds of evidence in 21 HEPA policies and interviewed 86 key policymakers involved in the policies. Qualitative content analysis was conducted on both policy documents and interview data.

**Results:**

Research evidence was mostly used to justify the creation of HEPA policies and, generally, implicitly without citation. The policies analysed used many types of evidence other than citable research. The evidence used in HEPA policies was found to fall into the following categories: societal framework, media, everyday knowledge and intuition, research evidence, and other types of evidence.

**Conclusions:**

Research evidence seems to be the only type of evidence used in policymaking. Competition between the use of other types of evidence and research evidence is constant due to the various sources of information on the Internet and elsewhere. However, researchers need to understand their role in translating research evidence into policymaking processes.

## Background

The health benefits of physical activity (PA) are supported by research evidence as well as the international public health community and its policies, including the World Health Organization (WHO) [1]. Regular PA that is sufficiently above baseline activity to produce health gains is referred to as health-enhancing PA (HEPA). Benefits of HEPA include reductions in obesity, coronary heart disease and stroke, diabetes, hypertension, colon cancer, breast cancer, and depression [2–4]. The integration of PA as part of everyday life has been shown to be beneficial, especially for people with a sedentary lifestyle [5–7]. The importance of a PA policy at national and local level to promote population-based PA is accentuated [3]. Criteria for developing and writing successful PA policies, action plans, and recommendations have also been identified [8, 9] and include highly consultative processes, such as multi-strategic, multi-level, and cross-sector approaches; public-private partnerships; clear identities, roles and timeframes; definitions of national guidelines and recommendations for HEPA; and policy development with evidence-informed processes. There are two terms for distinction: ‘evidence’ is used for the best available research evidence (in terms of quality and/or feasibility) and ‘other kinds of evidence’ is used to guide policy decisions such as expertise and contextual priorities, values, and resources [10]. The gaps seen between the use of research evidence and policy have been reported to include the different ways of using research evidence in policymaking by researchers and actual policymakers [11], availability of user-friendly data repositories for research evidence [12], development of dialogues and guidelines for policy briefs [13], and training for decision-makers [14]. Some policies and policymaking processes may therefore be particularly well informed by research evidence compared with others. Research evidence is considered only one of many influential factors for policymakers, who often base their choices on politics, values, and experiences [15]. Nevertheless, institutional structures and mechanisms should be ensuring effective and appropriate use of evidence in health policy and practice [16].

However, there are strong economic and moral, and occasionally political, arguments for making better use of research evidence in policymaking [17]. Economic arguments try to ensure that public investment in research is wisely spent by maximising use and helping to identify cost-effective policy decisions based on sound evidence. Moral arguments try to use the best possible evidence of positive outcomes to maximise benefits when people’s lives are intervened in, through, for example, schooling or social and healthcare services. Political arguments respect public expectations of the use of research evidence in areas such as medical interventions, making it important for politicians to at least appear to be using research evidence [17]. Systematic assessment of the use of research evidence in HEPA policies could consequently support the translation of research into policymaking structures, networks, and institutional arrangements relevant to HEPA.

Previous international research on HEPA policies [18–22] has leaned towards country-specific descriptive case studies or decontextualized systematic reviews of HEPA policies, neither of which yields a firm conclusion on how research evidence and other types of evidence have been used to influence policy approaches and foreseen outcomes. Based on research conducted previously [8, 18], the development principles of a policy should be followed more closely to increase the effectiveness of the preparation and implementation of HEPA policies. Moreover, process evaluation, monitoring, accountability, implementation, further interaction between stakeholders, including a cross-sector approach, and use of research evidence have been found to be insufficient in HEPA policies [19–22].

Institutions, interest groups, and networks interact with individuals and their ideas at different times and places during the policymaking process [23]. In policymaking, institutions form structures and norms for policymaking [24]. The institutions usually base their choices on rationality, organisational structures, or historical background, which opens a possibility for individual actors to influence policymaking [25]. If ideas lead the policymaking, the salience of argumentation, discourse, and advocacy become important in the policy process [26]. Networks of stakeholders in policymaking therefore create political associations and links between issues, which may explain policy stability and variations in time [23]. The process of policymaking consists of various stages of problem identification and issue recognition, and policy formulation, implementation, and evaluation [27]. Policy development is described as a continuous process of initiation, adoption, implementation, evaluation, and reformulation, but not necessarily a linear social and political process [28]. Research evidence or other types of evidence can therefore enter policymaking processes at any point, which should be considered when undertaking policy development and analysis.

This study is part of the EU-funded project Research into POlicy to enhance Physical Activity (REPOPA), with the overall aim of integrating scientific research knowledge, expertise, and real world policymaking processes. This could increase synergy and sustainability in promoting health and preventing disease, and promote physical activity in structural policymaking through different research and networking activities. The aims of the present article are to explore the use of research evidence in HEPA policies, identify when research evidence was used, and indicate which other types of evidence were used in HEPA policymaking. The study may be considered valuable in providing information on effective translation of research evidence into HEPA policymaking processes, which would ultimately lead to more effective HEPA policies.

## Methods

Each research team in the partner countries suggested relevant HEPA policies for consideration. Prior to the selection of HEPA policies for the study, the research team, e.g. principal researchers in each partner country, looked at the objectives, primary stakeholders involved, subpopulation specificity of policies, processes leading to release of the policies, and selection of documents used for the development of the policies. The policies were then categorised as national, regional, or local. Based on this primary inventory and in order to focus on the most recent developments, documents were selected according to the criteria of policies focusing on HEPA, being published by public authorities and representing HEPA policies in force at the beginning of 2012. The selected national, regional, and local HEPA policies ensured at least some variation in the scale and topic of the expected policy change: from major changes, such as governance structures and legislative perspective, to minor changes such as programmes, advice services, or building playgrounds. Some of the national, regional, and local policies belong to a ‘package’ of national, regional, or local policies created after elections for respective development purposes. They therefore also reflect wider policymaking processes and possibly the use of a wide base of research evidence. In the case of ‘package’ policies, the study concentrated on the specific policy documents for HEPA issues only. For the final set of HEPA policies, 21 policies from six European countries were selected with variation across the types of policies and policymaking processes. The topics of the sample of HEPA policies included in the analysis contained public health aspects (prevention, health promotion, and nutrition), HEPA aspects (sport, movement for health and sport for all, Olympics), infrastructure (transport, walking and cycling, sports halls and gyms), and places for PA (youth, schools, and neighbourhoods) [29]. The study included countries which already had an evidence-informed approach as normal practice (England), under development (Finland and the Netherlands), and similar to normal practice (Denmark, Italy, and Romania). The second criterion was that different regions within the European Union had to be represented since the project was funded by the Seventh Framework Programme of the European Commission.

To assess the use of research evidence in HEPA policies, content analyses of policy documents and semi-structured interviews were used. The content analyses of the policy documents and the interviews complemented each other to identify the various ways of using evidence in policymaking. The intention was to facilitate comparisons between countries in the use of research evidence in policymaking and take into consideration contextual differences through interviews. In each country, a policy was identified along with the content, stakeholders, and processes in relation to the relevant research. A series of questions was put to the stakeholders and policymakers to recall the use of research evidence. Further questions were allocated depending on the references to the use of research evidence in speeches, statements, guidelines, and similar background documents.

The HEPA policy process was split into agenda-setting and policy development phases in order to identify the use of research evidence at different stages of the policymaking process on various topics and issues. The agenda-setting phase and the use of research evidence were mostly traced through interviews and the use of research evidence in the policymaking process through policies or other supporting documents. The agenda-setting phase and the use of research evidence were dependent on interviews, as most of the countries did not have records on the decisions made in the agenda-setting phase, whereas the policymaking phase was often described in policies or related background documents.

### Content analysis of policy documents

Based on the literature review for the project proposal and its update upon acceptance of funding for the use of research evidence in policymaking, the study was undertaken using a qualitative descriptive approach inspired by political sciences [30, 31], public health sciences [15, 32, 33], and the multidisciplinary field of knowledge transfer, knowledge utilisation, and lesson learning [34–36]. In many policy processes, colleagues or areas of expertise are also commonly used for evidence-informed decisions and policymaking [37, 38]. Given the complex processes of policymaking, content analysis of policy documents and stakeholder interviews was selected to find how tacit (unspoken), implicit, and indirect knowledge and opinions shaped the policymaking processes and how more explicit and particular use of research evidence was integrated into policymaking processes.

The content analysis of HEPA policy documents followed the ideas of Ritchie and Spencer [39] and consisted of the process of analysing policy documents by issues and topics with the help of a set of guiding questions. After mapping the issues and topics, the HEPA documents were further analysed to establish type and use of evidence for each policy. Each HEPA policy was reread, sifted, charted, and sorted according to the key issues, topics and themes, confirming the patterns of research and other evidence used, and the research evidence was categorised into various types of evidence used. When research evidence was cited, the content was analysed to establish how the citation supported the policy statements or position in addressing the issues. The citable research considered consisted of journal articles, book chapters, and working papers and reports typically produced by research institutes, universities, and other independent research units. Other types of evidence considered were readings, media including the Internet and news, interactions with peers or stakeholders, and the involvement of participants in hearings, working groups, meetings, and the like.

Where appropriate, a published protocol and tool developed by Lavis et al. [15] and Hanney et al. [11] were used to identify, review, and locate the explicit use of research evidence in policy documents through content analysis. The REPOPA research team, lead by the work package leader, prepared a common guideline for all partners for the content analysis of policy documents and to indicate the information to be retrieved from the selected policy documents. Thereafter, the findings were reported in English and summarized into one report. The implementation phase of the policies was not included in the analysis, since this lies beyond the scope of the present study.

The common guideline that was developed covered the criteria for the selection of policies, theoretical models of the policymaking phases based on Kingdon [26], the focus of analysis in relation to topics, goals, and processes in the form of thematic questions, identification of stakeholders, the process description of the policy analysis and instructions for the HEPA policy analysis of the role of evidence in policymaking based on Ritchie and Spencer [39], and a schematic example of the analysed text of a policy.

### Semi-structured interviews, interview guide, and topics covered

During the HEPA policy document analysis, key informants for each policy were identified for face-to-face semi-structured interviews in order to verify the findings of the content analysis stage and gather information on any gaps in the uncited or implicit use of research evidence. The purposeful sampling of 87 informants in six countries was based on the selection criteria of the interviewees being directly involved in the policymaking process and able to report on the use of research evidence or other kinds of evidence in the policymaking process. In each policy, one of the interviewees was employed at the organisation responsible for the policy. The interviewees had been involved in policymaking processes of the policy on which they were interviewed and were policymakers, researchers, public sector officers, or other influential stakeholders. All the interviewees were contacted by email or phone by the research team in each country with basic information on the project and consent forms in the local language. The interviews were conducted by research team members with backgrounds in health and social sciences in the local language, recorded when accepted and transcribed for the analysis. An interview guide was developed by the REPOPA team led by the work package leader. In the guide, the questions for the policymaking process were split into agenda-setting and policy development phases. The questions followed the protocols [11, 15] with adaptation to the context and gaps in information after document analysis. A consent form, description of the research project, and preliminary list of questions were also provided. The interview questions were based on the analysis of the policy documents, especially the gaps found in terms of the evidence used. They followed the same structure, topics, and issues. The interviews were conducted after the document analysis of the policies.

To adapt the interviews for each context, each country team conducted between one and three pilot interviews to modify the questions, interview process, and language.

The semi-structured interviews were selected as a method to gain complementary information and to facilitate the adaptation of questions to each policy and case. The stakeholder interviews for each policy verified facts identified during the content analysis of policy documents, gaps in the information gathered in the use of research evidence, and the needs of policymakers to use evidence in the policymaking process.

In the semi-structured interviews, the interviewees were asked to recall the policymaking process period, review their files before the interview, and explain the policymaking process and the parties involved. The main issues in the interviews were how and why research evidence and other types of evidence did or did not enter into the policymaking, as well as the origin, influence, and characters of policymaking. The topics covered included policy changes, their significance, the use of research evidence and other types of evidence in bringing the content and change into the policy, the factors describing how and why the issues appeared on the policy agenda, and the factors influencing the way the policy developed.

### Analysis of research evidence in policy documents and interviews

The selected HEPA policy documents and interviews were mapped, coded, and further analysed using the interview questions as guidance. Each HEPA policy and transcribed interview was reread, coded, sifted, charted, and sorted according to the key issues and themes, confirming the patterns of use of research and other evidence, and categorised into various topics and themes. The interviews were analysed by the country-based research teams using an interpretative approach derived from a content analysis of the policy [38]. The coding was performed by the interviewers, and the accuracy of the content analysis was supported by independent assessments by the team members in the country research teams. The data from the interviews were enriched by the policy document analysis, and sometimes with additional available documentation, such as speeches, statements, guidelines, and similar background documents. However, in most cases, additional documents were not available and thus the overall analysis of the use of research evidence relied on data obtained from the analysed policy documents and interviews.

The policies and interviews were further analysed to identify the use of evidence to support the policies. For the distinction between research evidence and other types of evidence, a list of evidence with modifications was developed based on Lavis, Ross, and Hurley [15]. Evidence was considered as:

- Demographic and statistical data (facts and reports used as a background or for prioritising policy areas)
- Non-systematically derived peer-reviewed scientific literature (an ad hoc search for research articles or other similar materials that are not systematically assessed and applied)
- Systematically derived peer-reviewed scientific literature (literature derived from a systematic literature search in databases, reviewed, summarised, and contextualised to a specific policy context)
- International, transnational, national, regional, or local standards and guidelines
- Knowledge derived from community consultations, stakeholder workshops, and in-house consultations
- National reports (national reports on urban development and HEPA or other reports)
- Knowledge derived from expert consultations or policy briefs developed by research institutions

In the content analysis stage, the policy documents were reviewed to identify the explicit use of research evidence. When research evidence was cited, the content was analysed to establish the way the citation supported the policy statements or position in addressing the issues. The research evidence was published as journal articles, book chapters, working papers, or reports typically produced by research institutes, universities, or other independent research units. Other types of evidence referred to readings, information from media, and interaction with peers or stakeholders. Involvement of people in hearings, working groups, meetings, and the like was also looked for in the policy documents and interviews.

Finding patterns when research was used required a framework for determining the contexts in which policymaking occurred [11, 27, 40]. In accordance with Bowen et al. [40], we identified factors influencing policymaking, such as ideas, ideological or political values, and interests and institutions from the policy documents and interviews. The content analysis of HEPA policies and interviews detected and defined these items as evidence used in the HEPA policy development process, e.g. the content analysis of the document and interview themes categorised and classified them and looked for evidence of use: what constituted research evidence or other kinds of evidence in HEPA policy?

## Results

### The use of research and other types of evidence in policymaking

The interviews complemented the understanding of the use of research evidence in the agenda-setting and actual policymaking phases of HEPA policies. Policy documents [29] provided information on the actual use of research evidence, whereas interviews provided other types of information. The usual factors influencing policymaking were ideas, interests, and institutions; these were further subdivided to specify the influencing factors more precisely. This is especially important due to the increasing influence of the media, including social media and media in general, on policymaking.

By contrasting and searching for internal patterns, connections, and explanations for themes, categories, or issues in the content analysis, it was possible to describe the HEPA policymaking process, involvement of stakeholders and use of research evidence, and other types of evidence for judging HEPA policies for, for example, various subgroups. Sometimes, research evidence on health determinants and efficacy of interventions was used to legitimise actions, with other types of evidence used to accommodate contextually salient factors such as culture, community and organisational values, resources, and political priorities, which defined the usefulness of evidence for HEPA policymaking and implementation.

Explicit citable use of research evidence was mostly found in the justification of HEPA policies and when stating a specific study or publication as a trigger for a policy. It seemed that the visions and goals of the policies were not validated by research evidence but by other types of evidence. For the justification of the policies, instead of using peer-reviewed scientific articles, the policymakers used various types of secondary publications, such as national and international reports and recommendations. In other parts of the policy, such as target setting or actions for target groups, explicit research evidence was rarely used.

Neither policy document analysis nor interviews provided information on whether explicit or implicit evidence or citable research evidence was really influencing policies or whether implicit evidence was informed by research evidence.

The study found that most countries did not have routine reporting mechanisms for policy decisions during policymaking processes that would have been accessible for this study. Some countries had records available in the form of media communications, lists of members of the various committees, working groups or legislative commissions, and report databases. Theoretically, in the agenda-setting phase, priorities are set out for the policymaking process and in the actual policymaking development phase the research evidence should support the issues to be considered, chosen, and selected as part of the policy. Nevertheless, in the studied HEPA policies, the decisions made in the agenda-setting and policymaking phases were not recorded and therefore the information on the use of research evidence was based on what was found in the documents and their references and in the recall of interviewees.

### Evidence used in HEPA policymaking

Where research evidence was used, it was identified in an ad hoc manner in the policymaking phase and consisted of epidemiological research, population studies or statistics, and case studies. Peer-reviewed research articles and research based on surveys, as well as monitoring, evaluation, and implementation studies were rarely used. When the HEPA policies used citable research evidence, it was not necessarily peer-reviewed scientific articles. Instead, various types of national and international reports and recommendations were used. In most cases, when paragraphs in the policy documents suggested being informed by scientific knowledge, explicit research evidence was seldom referenced.

**Figure 1:**
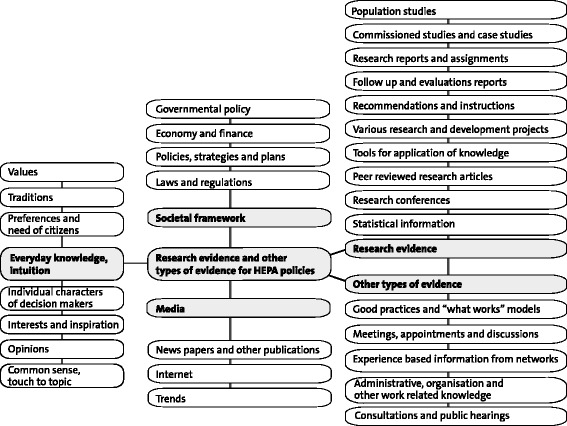
Research evidence and other type of evidence used in HEPA policymaking (in separate file).

According to the interviews, the research evidence used in HEPA policymaking was based on previous strategies, programmes, recommendations, guidelines, alignments, traditions, political support, trends, legislation, or economics (Figure 1). In the interviewees’ opinions, policies used previous strategies, and the continuation of the former policy processes made policies to some extent depend on and follow the previous policies. In addition, the interviewees claimed that lessons learnt from other projects or interventions were seldom used as evidence for policymaking.

Policymakers obtained other types of evidence from experiences, such as earlier national or international good practices, projects or programmes, or information gathered from personal networks (Figure 1). Meetings, seminars, and workshops were also used as experience-based information and evidence for policymaking. There was a trend to use expert or public consultation as evidence for policymaking. As most of the policies were prepared by committees or working groups, the members brought knowledge, experiences, practices, networks, and values to the policymaking. This other type of evidence was labelled as common sense and intuition, which often directed the selection of topics, discussions, and decisions in policymaking processes. Moreover, the interviewees reported that individual and socially structured factors such as values, interests, and common sense, including traditions and personal inspiration, influenced policymaking. In addition, ideological and political values of members of working groups and committees, such as political parties and their programmes, democratic engagement of citizens, or specific advocacy for certain population groups by the representatives of organisations, influenced policymaking (Figure 1).

The most challenging part of the different types of evidence used in the policymaking was the use of the Internet and media information for policymaking. The challenge was even stronger when the policymaking process had occurred some years ago, which was the case for most of the policies. According to the interviews, for the political level of policymaking, the media and its attention to certain topics during the policymaking process seemed to be an important source and legitimation of information and concern for taking up the issue in the policy.

Based on the content analysis of policy documents and interviews, the types of evidence used were listed, the items from which were classified and categorised; these five broad categories, namely research evidence, other types of evidence, societal frameworks, media, and everyday knowledge and intuition, are depicted in Figure 1.

## Discussion

The aims of the study were to assess how, to what extent, and where research evidence and other evidence were used in HEPA policymaking. In this qualitative study of the content analysis of 21 policy documents and 86 stakeholder interviews from six European countries, it was found that most countries did not have routine reporting mechanisms for policy decisions using research evidence during the policymaking processes. This can be considered a weakness in the transparency of the policymaking processes. Other related reports were available, but they were not necessarily related to policies or policymaking processes as such, they did not reflect the chosen policies, or particularly explain, describe, or confirm the use of specific research evidence in policymaking. Overall, from the content analysis of the policy documents, it was established that there was a lack of citable research evidence use in policy documents as it was rarely explicitly expressed. This was also confirmed by the interviews with stakeholders involved in policymaking. It was found that implicit evidence, such as common knowledge, facts, and practices, were primarily used in the policies.

However, when investigated in more depth, the study proved that the use of other types of evidence and broader societal framework information (both of which may or may not have been informed by citable research) was common. The use of non-citable research evidence was difficult to define, as many guidelines, standards, and recommendations by international and national organisations are based on citable research but without explicitly showing the citations used. In addition, public hearings and experiences from other sectors and colleagues from networks as other types of evidence confirmed the expectation that various types of evidence were used in policymaking. As shown in a previous study [41, 42], the policy dialogue represents an evolving approach for the use of research evidence in policymaking. Nevertheless, these political interactions could be more evidence-informed and need evaluations. The policy dialogue requires a range of dissemination means and formats to be adopted for particular issues and types of contexts [43].

The content analysis approach showed that research evidence seems to be only one type of information used in policymaking, and competition between the use of other types of information and research evidence is constant due to various sources of information on the Internet and elsewhere. To navigate the use of evidence in policy and practice, it is therefore necessary to understand how ideas spread through systems, how decisions and policies are made, and how capacity is required to use evidence in policymaking [40]. The use of research evidence and being informed about it seemed to be particularly important issues in the present policymaking at EU and national levels, due to reforms of the systems of research institutes and their funding in, for example, Finland. However, in general, the use of research evidence seems to depend on close contacts with researchers as well as values given to accept or reject the evidence related to a policy, which often means that research that supports a policymaker’s own views will be taken into consideration [43]. Based on earlier studies [39], in the policymaking process, the use of research and other types of evidence is influenced by the positions of stakeholders and existing institutional arrangements at national, regional, and local levels.

Evidence-informed policymaking is a contingent, complex system-like, non-linear, and emergent process of producing, managing, and implementing new knowledge. Most authors appear to agree with at least the possibility that evidence-informed policymaking can work as a virtuous cycle in order to improve policymaking, even if there are widely divergent ideas about what constitutes the component parts of the evidence-informed policymaking process, how this can and should be done, and what it should achieve [44]. Policymakers and other stakeholders in HEPA policymaking should therefore clearly face, solve, and manage the use of research evidence in policymaking and realise true evidence-informed policymaking. In this study, various secondary publications were used for policymaking, as this form fits policymakers and information is filtered and often judged to fit the context. However, the explicit research evidence may not be relevant for target setting or actions for target groups since local priorities and contexts need to be taken into consideration.

The aim of the study, based on successive phases of policy analysis and stakeholder interviews, was to allow more in-depth identification of evidence use for HEPA policymaking and of the pattern of evidence use. One major limitation of this study was that the interviewees did not recall the policymaking process in detail, even if the policies were still in force. The evidence used in the policymaking process also remained unclear to some extent despite selection of the most recent HEPA policies to minimise recall bias. However, this study clearly shows that there is a need for future policy research to take into account the various types of evidence used in policymaking and to choose a study design that can minimise recall bias. A further study area is the use of the Internet and its sources of research evidence and other types of evidence in policymaking. Additional countries would have increased the validity of the results and a variety of approaches to HEPA and the use of research evidence. However, the selected policies were not necessarily from the same levels due to the non-existence or unavailability of similar policies across the six countries.

## Conclusions

The use of research evidence identified through this study will be relevant to other researchers and research units working with policymakers at national, regional, and local levels. Similarly, the findings yield useful approaches for consideration by policymakers who work with researchers in academic institutions. These research-policy nexuses are clearly intended for practical applications in policymaking. Not all research is meant for this purpose. Research evidence seems to be only one type of information used in policymaking. Competition between the use of other types of information and research evidence is constant due to various sources of information on the Internet and elsewhere. In addition to the research evidence, other types of evidence from good practices, experiences from networks, work-related knowledge, and public hearings were used in policymaking; for example, values, traditions, interests, and opinions also influence everyday knowledge for policymaking. Moreover, past policies, regulations, and other policies influence and are used to frame policies. However, the use of research evidence seems to depend on close contacts between policymakers and researchers as well as on easy availability of and access to information. Making better use of research evidence in developing HEPA policies requires researchers to understand their role in policymaking and in translating research into policymaking [44].

Consolidating and revising HEPA policies to be more informed by research evidence at national, regional, or local level would strengthen systematic assessment of structures and institutional arrangements of HEPA policies and make them more effective. HEPA policies are embedded within a wider socio-economic context, and HEPA policies should therefore target wider determinants of physical activity and its impact on health. Using more explicit research evidence in policymaking could also offer alternatives and options for research evidence in more transparent ways.

## Abbreviations

HEPA: Health-enhancing physical activity
PA: Physical activity
REPOPA: REsearch into POlicy in Physical Activity
